# 4-{[2-(2,4-Dinitro­phen­yl)hydrazinyl­idene]meth­yl}phenol ethanol hemisolvate

**DOI:** 10.1107/S1600536810045782

**Published:** 2010-11-13

**Authors:** Xiu-Rong Zhai

**Affiliations:** a Department of Chemisry and Chemical Engineering, Jining University, 273155 Qufu, Shandong, People’s Republic of China

## Abstract

In the title compound, C_13_H_10_N_4_O_5_·0.5C_2_H_5_OH, the two benzene rings form a dihedral angle of 4.29 (9)°. The ethanol solvent mol­ecule was treated as disordered between two orientations related by symmetry (center of inversion), with occupancies fixed at 0.5. The crystal packing, stabilized by inter­molecular O—H⋯O and N—H⋯O hydrogen bonds and π–π inter­actions [indicated by the short distance of 3.7299 (7) Å between the centroids of benzene rings from neighbouring mol­ecules], exhibits short inter­molecular O⋯O contacts of 2.8226 (3) Å.

## Related literature

For related structures, see: Baughman *et al.* (2004 ▶); Shi *et al.* (2008 ▶); Ji & Shi (2008 ▶). For bond-length data, see: Allen *et al.* (1987 ▶).
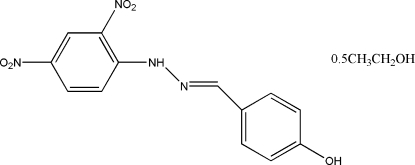


## Experimental

### 

#### Crystal data

C_13_H_10_N_4_O_5_·0.5C_2_H_6_O
*M* *_r_* = 325.28
Triclinic, 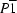

*a* = 7.0935 (16) Å
*b* = 7.2888 (17) Å
*c* = 14.458 (3) Å
α = 100.156 (4)°
β = 96.378 (4)°
γ = 100.604 (4)°
*V* = 715.2 (3) Å^3^
*Z* = 2
Mo *K*α radiation
μ = 0.12 mm^−1^
*T* = 295 K
0.15 × 0.12 × 0.10 mm

#### Data collection

Bruker SMART APEXII CCD area-detector diffractometer
Absorption correction: multi-scan (*SADABS*; Bruker, 2005 ▶) *T*_min_ = 0.982, *T*_max_ = 0.988
4065 measured reflections
2776 independent reflections
1543 reflections with *I* > 2σ(*I*)
*R*_int_ = 0.020

#### Refinement

*R*[*F* ^2^ > 2σ(*F* ^2^)] = 0.064
*wR*(*F* ^2^) = 0.230
*S* = 1.04
2776 reflections
227 parameters
3 restraints
H-atom parameters constrained
Δρ_max_ = 0.46 e Å^−3^
Δρ_min_ = −0.25 e Å^−3^

### 

Data collection: *APEX2* (Bruker, 2005 ▶); cell refinement: *SAINT* (Bruker, 2005 ▶); data reduction: *SAINT*; program(s) used to solve structure: *SHELXTL* (Sheldrick, 2008 ▶); program(s) used to refine structure: *SHELXTL*; molecular graphics: *SHELXTL*; software used to prepare material for publication: *SHELXTL*.

## Supplementary Material

Crystal structure: contains datablocks global, I. DOI: 10.1107/S1600536810045782/cv2785sup1.cif

Structure factors: contains datablocks I. DOI: 10.1107/S1600536810045782/cv2785Isup2.hkl

Additional supplementary materials:  crystallographic information; 3D view; checkCIF report

## Figures and Tables

**Table 1:** Hydrogen-bond geometry (Å, °)

*D*—H⋯*A*	*D*—H	H⋯*A*	*D*⋯*A*	*D*—H⋯*A*
N1—H1⋯O2	0.89	1.97	2.605 (3)	127
O6—H6*A*⋯O3	0.85	1.86	2.702 (9)	174
O5—H5⋯O6^i^	0.85	2.26	2.882 (9)	130
O5—H5⋯O4^ii^	0.85	2.47	3.116 (4)	133
O5—H5⋯O3^ii^	0.85	2.60	3.404 (4)	159
N1—H1⋯O2^iii^	0.89	2.63	3.449 (4)	153

Symmetry codes: (i) 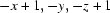; (ii) 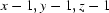; (iii) 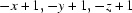.

**Fig. 1.**
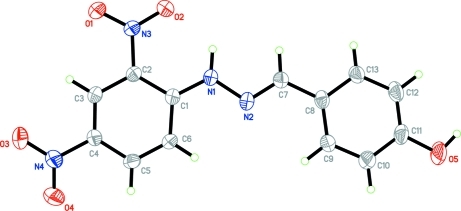
View of the title compound showing the atomic labeling and 30% probability displacement ellipsoids. Solvent molecule omitted for clarity.

## supplementary crystallographic information

### Comment

As a contribution to structural studies of 2,4-dinitrophenylhydrazones (Baughman *et al.*, 2004; Shi *et al.*, 2008; Ji *et al.*, 2008;) we present here the crystal structure of the title compound (I).

In (I) (Fig. 1), all bond lengths and angles are normal (Allen *et al.*, 1987). The crystal packing, stabilized by intermolecular O—H···O and N—H···O hydrogen bonds (Table 1) and π–π interactions proved by short distance of 3.7299 (7) Å between the centroids of benzene rings from the neighbouring molecules, exhibits short intermolecular O···O contacts of 2.8226 (3) Å.

### Experimental

The title compound was synthesized by the reaction of (2,4-dinitro-phenyl)-hydrazine (1 mmol, 198.1 mg) with 4-hydroxy-benzaldehyde (1 mmol, 122.1 mg) in ethanol (20 ml) under reflux conditions (338 K) for 4 h. The solvent was removed and the solid product recrystallized from ethanol. After five days brown crystals were obtained that were suitable for X-ray diffraction study.

### Refinement

All H atoms were placed in idealized positions (C—H = 0.93–0.97 Å, N—H = 0.86 Å) and refined as riding atoms. For those bound to C, *U*_iso_(H) = 1.2 or 1.5*U*_eq_(C). while for those bound to N, *U*_iso_(H) = 1.2 *U*_eq_(N). The ethanol solvent molecule has been treated as disordered between two orientations related by symmetry (center of inversion) with occupancies fixed to 0.5 and with C—O, C—C and O···C distances restrained to 1.40 (1), 1.45 (1) and 2.40 (1) Å, respectively.

### Crystal data

**Table:**

C_13_H_10_N_4_O_5_·0.5C_2_H_6_O	*Z* = 2
*M**_r_* = 325.28	*F*(000) = 338
Triclinic, *P*1	*D*_x_ = 1.510 Mg m^−^^3^
Hall symbol: -P 1	Mo *K*α radiation, λ = 0.71073 Å
*a* = 7.0935 (16) Å	Cell parameters from 748 reflections
*b* = 7.2888 (17) Å	θ = 2.9–24.3°
*c* = 14.458 (3) Å	µ = 0.12 mm^−^^1^
α = 100.156 (4)°	*T* = 295 K
β = 96.378 (4)°	Block, brown
γ = 100.604 (4)°	0.15 × 0.12 × 0.10 mm
*V* = 715.2 (3) Å^3^	

### Data collection

**Table:**

Bruker SMART CCD area-detector diffractometer	2776 independent reflections
Radiation source: fine-focus sealed tube	1543 reflections with *I* > 2σ(*I*)
graphite	*R*_int_ = 0.020
phi and ω scans	θ_max_ = 26.1°, θ_min_ = 1.5°
Absorption correction: multi-scan (*SADABS*; Bruker, 2005)	*h* = −7→8
*T*_min_ = 0.982, *T*_max_ = 0.988	*k* = −8→7
4065 measured reflections	*l* = −14→17

### Refinement

**Table:**

Refinement on *F*^2^	Secondary atom site location: difference Fourier map
Least-squares matrix: full	Hydrogen site location: inferred from neighbouring sites
*R*[*F*^2^ > 2σ(*F*^2^)] = 0.064	H-atom parameters constrained
*wR*(*F*^2^) = 0.230	*w* = 1/[σ^2^(*F*_o_^2^) + (0.1117*P*)^2^ + 0.1836*P*] where *P* = (*F*_o_^2^ + 2*F*_c_^2^)/3
*S* = 1.04	(Δ/σ)_max_ < 0.001
2776 reflections	Δρ_max_ = 0.46 e Å^−^^3^
227 parameters	Δρ_min_ = −0.25 e Å^−^^3^
3 restraints	Extinction correction: *SHELXTL* (Sheldrick, 2008), Fc^*^=kFc[1+0.001xFc^2^λ^3^/sin(2θ)]^-1/4^
Primary atom site location: structure-invariant direct methods	Extinction coefficient: 0.017 (7)

### Special details

**Table:**

Geometry. All e.s.d.'s (except the e.s.d. in the dihedral angle between two l.s. planes) are estimated using the full covariance matrix. The cell e.s.d.'s are taken into account individually in the estimation of e.s.d.'s in distances, angles and torsion angles; correlations between e.s.d.'s in cell parameters are only used when they are defined by crystal symmetry. An approximate (isotropic) treatment of cell e.s.d.'s is used for estimating e.s.d.'s involving l.s. planes.
Refinement. Refinement of *F*^2^ against ALL reflections. The weighted *R*-factor *wR* and goodness of fit *S* are based on *F*^2^, conventional *R*-factors *R* are based on *F*, with *F* set to zero for negative *F*^2^. The threshold expression of *F*^2^ > σ(*F*^2^) is used only for calculating *R*-factors(gt) *etc*. and is not relevant to the choice of reflections for refinement. *R*-factors based on *F*^2^ are statistically about twice as large as those based on *F*, and *R*- factors based on ALL data will be even larger.

### Fractional atomic coordinates and isotropic or equivalent isotropic displacement parameters (Å^2^)

**Table:**

	*x*	*y*	*z*	*U*_iso_*/*U*_eq_	Occ. (<1)
O1	0.6437 (4)	0.7313 (3)	0.73110 (16)	0.0700 (8)	
O2	0.4959 (4)	0.5632 (3)	0.59767 (16)	0.0683 (8)	
O3	0.7183 (5)	0.4849 (4)	1.00571 (18)	0.0973 (11)	
O4	0.5709 (5)	0.2032 (4)	1.00864 (18)	0.1019 (12)	
O5	−0.1603 (4)	−0.6843 (4)	0.20416 (18)	0.0890 (10)	
H5	−0.1738	−0.6648	0.1478	0.134*	
N1	0.3674 (3)	0.1977 (3)	0.57776 (16)	0.0470 (7)	
H1	0.3863	0.2907	0.5452	0.070*	
N2	0.2771 (3)	0.0174 (4)	0.53066 (17)	0.0479 (7)	
N3	0.5558 (4)	0.5794 (4)	0.68230 (18)	0.0499 (7)	
N4	0.6183 (5)	0.3288 (5)	0.9659 (2)	0.0698 (9)	
C1	0.4286 (4)	0.2316 (4)	0.6718 (2)	0.0412 (7)	
C2	0.5213 (4)	0.4143 (4)	0.7248 (2)	0.0420 (7)	
C3	0.5848 (4)	0.4436 (4)	0.8208 (2)	0.0474 (8)	
H3	0.6479	0.5642	0.8544	0.057*	
C4	0.5548 (5)	0.2961 (4)	0.8656 (2)	0.0493 (8)	
C5	0.4642 (5)	0.1133 (4)	0.8163 (2)	0.0511 (8)	
H5A	0.4453	0.0131	0.8485	0.061*	
C6	0.4040 (4)	0.0821 (4)	0.7223 (2)	0.0467 (8)	
H6	0.3450	−0.0405	0.6899	0.056*	
C7	0.2242 (4)	0.0014 (4)	0.4422 (2)	0.0473 (8)	
H7	0.2487	0.1092	0.4157	0.057*	
C8	0.1273 (4)	−0.1784 (5)	0.3812 (2)	0.0464 (8)	
C9	0.0970 (5)	−0.3493 (5)	0.4128 (2)	0.0551 (9)	
H9	0.1418	−0.3512	0.4755	0.066*	
C10	0.0018 (5)	−0.5148 (5)	0.3525 (2)	0.0612 (9)	
H10	−0.0189	−0.6280	0.3748	0.073*	
C11	−0.0638 (5)	−0.5156 (5)	0.2591 (2)	0.0585 (9)	
C12	−0.0313 (5)	−0.3488 (5)	0.2267 (2)	0.0617 (10)	
H12	−0.0734	−0.3487	0.1635	0.074*	
C13	0.0629 (4)	−0.1817 (5)	0.2868 (2)	0.0530 (8)	
H13	0.0838	−0.0693	0.2638	0.064*	
O6	1.0107 (12)	0.7495 (11)	0.9734 (7)	0.148 (3)	0.50
H6A	0.9238	0.6597	0.9822	0.222*	0.50
C14	0.9498 (18)	0.9229 (13)	0.9968 (12)	0.115 (5)	0.50
H14A	1.0102	0.9845	1.0602	0.138*	0.50
H14B	0.8128	0.8924	0.9983	0.138*	0.50
C15	0.965 (5)	1.042 (3)	0.9313 (12)	0.259 (14)	0.50
H15A	0.9230	1.1573	0.9555	0.388*	0.50
H15B	1.0979	1.0729	0.9223	0.388*	0.50
H15C	0.8861	0.9802	0.8717	0.388*	0.50

### Atomic displacement parameters (Å^2^)

**Table:**

	*U*^11^	*U*^22^	*U*^33^	*U*^12^	*U*^13^	*U*^23^
O1	0.1033 (19)	0.0395 (14)	0.0569 (14)	−0.0028 (12)	0.0025 (13)	0.0063 (11)
O2	0.0965 (19)	0.0546 (15)	0.0477 (14)	0.0023 (13)	−0.0027 (13)	0.0160 (11)
O3	0.137 (3)	0.078 (2)	0.0503 (15)	−0.0157 (18)	−0.0210 (16)	0.0019 (14)
O4	0.172 (3)	0.075 (2)	0.0533 (17)	0.008 (2)	−0.0009 (18)	0.0265 (15)
O5	0.111 (2)	0.0689 (18)	0.0581 (15)	−0.0248 (16)	−0.0010 (15)	−0.0114 (13)
N1	0.0544 (15)	0.0402 (14)	0.0404 (14)	0.0029 (11)	−0.0003 (11)	0.0045 (11)
N2	0.0490 (15)	0.0455 (15)	0.0426 (15)	0.0056 (11)	0.0013 (12)	−0.0009 (11)
N3	0.0627 (17)	0.0373 (15)	0.0473 (16)	0.0055 (12)	0.0062 (13)	0.0090 (11)
N4	0.102 (2)	0.057 (2)	0.0451 (17)	0.0100 (17)	−0.0015 (16)	0.0098 (15)
C1	0.0406 (15)	0.0407 (17)	0.0406 (16)	0.0074 (12)	0.0052 (12)	0.0049 (12)
C2	0.0451 (16)	0.0360 (16)	0.0441 (16)	0.0083 (12)	0.0062 (13)	0.0060 (12)
C3	0.0569 (19)	0.0370 (17)	0.0432 (17)	0.0064 (13)	0.0034 (14)	0.0003 (12)
C4	0.062 (2)	0.0447 (18)	0.0381 (16)	0.0085 (15)	0.0016 (14)	0.0058 (13)
C5	0.066 (2)	0.0380 (17)	0.0498 (18)	0.0065 (14)	0.0117 (15)	0.0129 (13)
C6	0.0561 (18)	0.0354 (16)	0.0456 (17)	0.0047 (13)	0.0054 (14)	0.0066 (13)
C7	0.0487 (17)	0.0467 (18)	0.0433 (17)	0.0051 (14)	0.0039 (14)	0.0080 (13)
C8	0.0381 (16)	0.056 (2)	0.0401 (16)	0.0039 (13)	0.0047 (13)	0.0020 (14)
C9	0.065 (2)	0.054 (2)	0.0402 (17)	0.0030 (16)	0.0031 (15)	0.0072 (14)
C10	0.071 (2)	0.054 (2)	0.054 (2)	0.0004 (17)	0.0107 (17)	0.0075 (16)
C11	0.055 (2)	0.058 (2)	0.0478 (19)	−0.0092 (16)	0.0056 (15)	−0.0063 (16)
C12	0.056 (2)	0.075 (3)	0.0406 (17)	−0.0040 (17)	−0.0054 (15)	0.0027 (16)
C13	0.0516 (18)	0.058 (2)	0.0431 (17)	0.0020 (15)	−0.0005 (14)	0.0091 (14)
O6	0.139 (7)	0.123 (7)	0.176 (8)	−0.003 (5)	0.043 (6)	0.034 (6)
C14	0.113 (9)	0.044 (5)	0.197 (16)	0.026 (5)	0.058 (10)	0.015 (7)
C15	0.35 (4)	0.24 (3)	0.20 (3)	0.10 (2)	0.06 (3)	0.01 (2)

### Geometric parameters (Å, °)

**Table:**

O1—N3	1.219 (3)	C7—H7	0.9300
O2—N3	1.227 (3)	C8—C13	1.385 (4)
O3—N4	1.229 (4)	C8—C9	1.390 (5)
O4—N4	1.210 (4)	C9—C10	1.368 (4)
O5—C11	1.356 (4)	C9—H9	0.9300
O5—H5	0.8500	C10—C11	1.376 (5)
N1—C1	1.346 (3)	C10—H10	0.9300
N1—N2	1.370 (3)	C11—C12	1.369 (5)
N1—H1	0.8900	C12—C13	1.372 (4)
N2—C7	1.270 (4)	C12—H12	0.9300
N3—O2	1.227 (3)	C13—H13	0.9300
N3—C2	1.439 (4)	O6—C14	1.407 (8)
N4—O3	1.229 (4)	O6—C15^i^	1.827 (19)
N4—C4	1.435 (4)	O6—H6A	0.8500
C1—C6	1.410 (4)	C14—C15^i^	1.11 (2)
C1—C2	1.413 (4)	C14—C14^i^	1.20 (2)
C2—C3	1.377 (4)	C14—C15	1.394 (9)
C3—C4	1.346 (4)	C14—H14A	0.9600
C3—H3	0.9300	C14—H14B	0.9600
C4—C5	1.394 (4)	C15—C14^i^	1.11 (2)
C5—C6	1.344 (4)	C15—O6^i^	1.828 (19)
C5—H5A	0.9300	C15—H15A	0.9601
C6—H6	0.9300	C15—H15B	0.9600
C7—C8	1.447 (4)	C15—H15C	0.9600
			
C11—O5—H5	105.5	C9—C10—C11	120.6 (3)
C1—N1—N2	119.9 (3)	C9—C10—H10	119.7
C1—N1—H1	120.9	C11—C10—H10	119.7
N2—N1—H1	119.2	O5—C11—C12	123.4 (3)
C7—N2—N1	114.8 (3)	O5—C11—C10	117.2 (3)
O1—N3—O2	121.8 (3)	C12—C11—C10	119.3 (3)
O1—N3—O2	121.8 (3)	C11—C12—C13	120.5 (3)
O1—N3—C2	119.3 (3)	C11—C12—H12	119.8
O2—N3—C2	119.0 (2)	C13—C12—H12	119.8
O2—N3—C2	119.0 (2)	C12—C13—C8	120.8 (3)
O4—N4—O3	122.2 (3)	C12—C13—H13	119.6
O4—N4—O3	122.2 (3)	C8—C13—H13	119.6
O4—N4—C4	119.2 (3)	C14—O6—H6A	108.9
O3—N4—C4	118.5 (3)	C15^i^—O6—H6A	109.4
O3—N4—C4	118.5 (3)	C15^i^—C14—C14^i^	74.4 (13)
N1—C1—C6	120.5 (3)	C15^i^—C14—C15	124.3 (13)
N1—C1—C2	122.8 (3)	C14^i^—C14—C15	49.9 (12)
C6—C1—C2	116.7 (3)	C15^i^—C14—O6	92.5 (13)
C3—C2—C1	121.3 (3)	C14^i^—C14—O6	124.7 (14)
C3—C2—N3	116.1 (3)	C15—C14—O6	117.0 (10)
C1—C2—N3	122.5 (3)	C14^i^—C14—H14A	65.2
C4—C3—C2	119.4 (3)	C15—C14—H14A	113.8
C4—C3—H3	120.3	O6—C14—H14A	108.3
C2—C3—H3	120.3	C15^i^—C14—H14B	112.3
C3—C4—C5	121.3 (3)	C14^i^—C14—H14B	127.7
C3—C4—N4	119.0 (3)	C15—C14—H14B	102.9
C5—C4—N4	119.7 (3)	O6—C14—H14B	107.2
C6—C5—C4	119.9 (3)	H14A—C14—H14B	106.8
C6—C5—H5A	120.0	C14^i^—C15—C14	55.7 (13)
C4—C5—H5A	120.0	C14^i^—C15—O6^i^	50.3 (8)
C5—C6—C1	121.3 (3)	C14—C15—O6^i^	90.5 (11)
C5—C6—H6	119.3	C14^i^—C15—H15A	81.5
C1—C6—H6	119.3	C14—C15—H15A	109.7
N2—C7—C8	122.5 (3)	C14^i^—C15—H15B	74.1
N2—C7—H7	118.8	C14—C15—H15B	108.2
C8—C7—H7	118.8	O6^i^—C15—H15B	93.5
C13—C8—C9	118.2 (3)	H15A—C15—H15B	109.5
C13—C8—C7	118.9 (3)	C14^i^—C15—H15C	165.6
C9—C8—C7	122.9 (3)	C14—C15—H15C	110.5
C10—C9—C8	120.5 (3)	O6^i^—C15—H15C	140.8
C10—C9—H9	119.7	H15A—C15—H15C	109.5
C8—C9—H9	119.7	H15B—C15—H15C	109.5

Symmetry codes:  (i) −*x*+2, −*y*+2, −*z*+2.

### Hydrogen-bond geometry (Å, °)

**Table:**

*D*—H···*A*	*D*—H	H···*A*	*D*···*A*	*D*—H···*A*
N1—H1···O2	0.89	1.97	2.605 (3)	127
O6—H6A···O3	0.85	1.86	2.702 (9)	174
O5—H5···O6^ii^	0.85	2.26	2.882 (9)	130
O5—H5···O4^iii^	0.85	2.47	3.116 (4)	133
O5—H5···O3^iii^	0.85	2.60	3.404 (4)	159
N1—H1···O2^iv^	0.89	2.63	3.449 (4)	153

Symmetry codes:  (ii) −*x*+1, −*y*, −*z*+1; (iii) *x*−1, *y*−1, *z*−1; (iv) −*x*+1, −*y*+1, −*z*+1.
